# Global Leukocyte DNA Methylation Is Associated with Dietary Methyl-Donor Intake and Cardiometabolic Risk in Rheumatoid Arthritis and Control Subjects

**DOI:** 10.3390/ijms27031578

**Published:** 2026-02-05

**Authors:** Gerardo A. Macias, Bertha Campos-López, Karen Pesqueda-Cendejas, Paulina E. Mora-García, Eneida Turiján-Espinoza, Juan M. Vargas-Morales, Isela Parra-Rojas, Ulises De la Cruz-Mosso

**Affiliations:** 1 Red de Inmunonutrición y Genómica Nutricional en las Enfermedades Autoinmunes, Departamento de Neurociencias, Centro Universitario de Ciencias de la Salud, Universidad de Guadalajara, Guadalajara 44340, Jalisco, Mexico; 2 Instituto de Neurociencias Traslacionales, Departamento de Neurociencias, Centro Universitario de Ciencias de la Salud, Universidad de Guadalajara, Guadalajara 44340, Jalisco, Mexico; 3 Programa de Doctorado en Ciencias en Biología Molecular en Medicina, Departamento de Biología Molecular y Genómica, Centro Universitario de Ciencias de la Salud, Universidad de Guadalajara, Guadalajara 44340, Jalisco, Mexico; 4 Programa de Doctorado en Ciencias de la Nutrición Traslacional, Departamento de Alimentación y Nutrición, Centro Universitario de Ciencias de la Salud, Universidad de Guadalajara, Guadalajara 44340, Jalisco, Mexico; 5 Laboratorio de Inmunología y Biología Celular y Molecular, Facultad de Ciencias Químicas, Universidad Autónoma de San Luis Potosi, San Luis Potosi 78000, San Luis Potosi, Mexico; 6 Departamento de Medicina Traslacional y Molecular, Centro de Investigación en Ciencias de la Salud y Biomedicina (CICSaB), Universidad Autónoma de San Luis Potosi, San Luis Potosi 78000, San Luis Potosi, Mexico; 7 Laboratorio de Investigación en Obesidad y Diabetes, Facultad de Ciencias Químico-Biológicas, Universidad Autónoma de Guerrero, Chilpancingo de los Bravo 39087, Guerrero, Mexico

**Keywords:** diet, epigenetic, autoimmunity

## Abstract

In rheumatoid arthritis (RA), altered DNA methylation patterns could be associated with pro-inflammatory, immune, and metabolic risk profiles. Notably, DNA methylation is dynamically regulated by the interplay of multiple factors, including diet, cardiometabolic status, and aging. Therefore, this study aimed to assess the associations between global leukocyte DNA methylation, dietary methyl-donor intake, and cardiometabolic risk in RA and control subjects (CS). A cross-sectional study was conducted with 123 female RA patients classified by the 2010 ACR-EULAR criteria, and 130 female CS. Leukocyte DNA methylation status was assessed with the 5-mC DNA ELISA Kit. RA patients exhibited significantly lower global DNA methylation levels than those with CS. RA status was independently associated with lower DNA methylation levels after adjustment for age and body mass index. Similarly, in both study groups methionine intake showed an independent inverse association with global DNA methylation across adjusted models and lower methylation levels were consistently associated with an unfavorable cardiometabolic profile, characterized by increased adverse adiposity- and lipid-related indexes. In conclusion, RA patients exhibited lower global leukocyte DNA methylation levels compared with CS. In both study groups, lower DNA methylation levels were associated with low methionine intake and an unfavorable cardiometabolic profile.

## 1. Introduction

Rheumatoid arthritis (RA) is a chronic autoimmune disease that primarily affects diarthrodial joints, characterized by synovial membrane inflammation and progressive joint destruction [1]. RA represents a major public health concern, with a prevalence ranging from 0.7% to 2.8% in the Mexican population, and it is predominant in women, with a female-to-male ratio of 3:1 [2,3].

Beyond joint involvement, RA is characterized by persistent systemic inflammation that acts as an independent driver of cardiovascular disease (CVD) risk and contributes to premature mortality [4]. Cardiovascular events account for nearly 40% of deaths in RA patients [5,6], with a CVD risk up to 3.6-fold higher than in the general population [7]. This excess risk is largely explained by immunometabolic dysregulation referred to as the “lipid paradox,” where chronic inflammation alters lipoprotein composition and function, rendering them atherogenic despite normal or reduced circulating lipid levels [8,9]. Consequently, dyslipidemia affects up to 60% of RA patients and is strongly associated with cardiometabolic complications and elevated CVD risk [10,11].

In this context, chronic inflammation and cardiometabolic alterations in RA may induce long-lasting changes in gene regulation, positioning epigenetic mechanisms as key contributors to disease pathophysiology. Among these, DNA methylation is a central epigenetic modification that occurs predominantly at CpG dinucleotides and plays a critical role in transcriptional regulation and genomic stability. Although once considered a relatively stable mark, DNA methylation is now recognized as a dynamic process that can occur at both gene-specific and global levels in response to environmental and metabolic cues [12,13].

Epigenetic regulation varies across cell types and is influenced by environmental exposures, metabolic status, and developmental stage [14,15]. Although gene-specific DNA methylation changes have been widely investigated in RA, fewer comparative studies have focused on global DNA methylation patterns in peripheral blood leukocytes. Available evidence indicates that RA patients exhibit global leukocyte DNA hypomethylation in peripheral blood mononuclear cells compared to control subjects (CS), a phenomenon that may contribute to aberrant immune activation and sustained inflammatory responses [16,17]. In parallel, hypomethylation of promoters of key pathogenic genes, including *IL6*, *STAT3*, *MMP1*, and *TBX5*, has been reported and correlates with their overexpression and systemic inflammation and joint damage [18,19,20,21]. Together, these findings support the relevance of both global and locus-specific epigenetic dysregulation in RA.

Notably, global leukocyte DNA methylation has been proposed as an integrative epigenetic marker that reflects the cumulative influences of genetic background, inflammation, metabolic status, and dietary exposures [22,23]. It provides a broader measure of epigenomic stability and one-carbon metabolic balance in chronic inflammatory conditions such as RA [24,25]. Accumulating evidence indicates that diet can modulate DNA methylation, representing a key molecular mechanism in disease prevention and health maintenance [26,27]. Thus, nutritional factors may play a central role in the interplay between epigenetic homeostasis, systemic inflammation, cardiometabolic alterations, and CVD risk. DNA methylation is particularly sensitive to the availability of S-adenosyl-methionine (SAM), the universal methyl donor generated through one-carbon metabolism, which depends on dietary intake of nutrients such as folate, methionine, choline, betaine, and B vitamins, including B6 and B12 [28]. Insufficient intake of these methyl-donor nutrients has been shown to modify global DNA methylation levels and the methylation status of disease-related gene promoters in both experimental models and human studies [12].

While global leukocyte DNA hypomethylation in RA has been linked to proinflammatory cytokine overexpression, aggressive synovial fibroblast phenotypes, and T-cell autoreactivity [17] no studies to date have evaluated in the Mexican RA population the combined relationship between dietary methyl-donor intake, global leukocyte DNA methylation, and cardiometabolic risk profiles. Therefore, the present study aimed to assess the associations between global leukocyte DNA methylation levels, dietary methyl-donor intake, and cardiometabolic risk in female patients with RA and CS from the Mexican population.

## 2. Results

### 2.1. General Characteristics

In this study, we identified that 87% of RA patients presented established disease (≥1 year of evolution), and 63% presented active disease status according to the DAS28-CRP score. When comparing CRP serum levels in RA patients vs. CS, significant differences were observed (4.6 mg/L vs. 0.87 mg/L; *p* < 0.001). Regarding RA pharmacological treatment, polypharmacy was identified in their treatment scheme, with methotrexate being the most prescribed (84%), followed by NSAIDs (69%) (Table 1).

### 2.2. Evaluation of Global Leukocyte DNA Methylation Status

**Figure 1:**
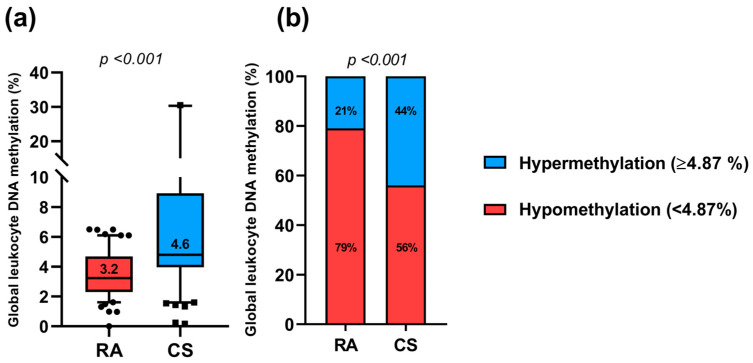
Global leukocyte DNA methylation in both study groups. (**a**) Medians of global leukocyte DNA methylation percentage from RA patients and CS, *p*-value obtained with the U Mann–Whitney test. Dots (RA) and squares (CS) indicate data outliers. (**b**) Global leukocyte DNA methylation percentage stratified into hypo (<4.87%) and hyper (≥4.87%) global methylation according to the tertile distribution of the obtained values; *p*-value obtained with the Chi-square test. RA: rheumatoid arthritis; CS: control subjects.

Regarding global leukocyte DNA methylation percentage in both study groups, RA patients showed a median value of 3.2% of global leukocyte DNA methylation compared to 4.6% in the CS (*p* < 0.001; Figure 1a). According to this, when we stratified the global leukocyte DNA methylation percentage in RA and CS, it was observed that 79% of the RA patients presented global DNA hypomethylation (<4.87%), while only 21% presented global DNA hypermethylation (≥4.87%). According to the CS, 56% showed global DNA hypomethylation while 44% presented global DNA hypermethylation (Figure 1b).

### 2.3. Relationship Between the Global Leukocyte DNA Methylation Status, Nutritional, and Cardiometabolic Variables in Both Study Groups

Regarding the intake of methyl-donor nutrients stratified according to the methylation percentage, we observed a similar pattern in both study groups, a trend of low intake of pantothenic acid (B5) in participants with hypomethylation compared to the hypermethylation group (80% vs. 68%; *p* = 0.05). Significantly, we observed in participants with hypomethylation status a low intake of methionine (68% vs. 54%; *p* = 0.03) (Table 2).

According to the body composition variables, participants with hypomethylation presented a higher prevalence of excess weight evaluated by abdominal circumference (58% vs. 33%; *p* < 0.001), significant higher BMI scores (62% vs. 30%; *p* < 0.001); likewise, excess body fat (50% vs. 25%; *p* < 0.001) (Table 2).

Subsequently, when evaluating biochemical variables, it was observed that participants with hypomethylation presented a higher frequency of altered glucose (≥100 mg/dL) compared to the hypermethylation group (24% vs. 8%; *p* = 0.01). Regarding the lipid profile, it was identified that the percentage of altered LDL-C (≥100 mg/dL) was significantly higher in the hypomethylation group (46% vs. 31%; *p* = 0.01). Notably, when analyzing cardiometabolic variables, it was found a high CVD risk pattern in the participants with hypomethylation characterized by hs-CRP values of CVD risk (≥1 mg/L) (75% vs. 52%; *p* < 0.001), CMI risk score (74% vs. 39%; *p* < 0.001), WHtR index risk score (59% vs. 31%; *p* < 0.001), CRP/Albumin index risk score (69% vs. 48%; *p* = 0.004), TyG index risk score (74% vs. 41%; *p* < 0.001), and VAI risk score (72% vs. 44%; *p* < 0.001). This suggests that global DNA hypomethylation is characterized by a pattern of adverse metabolic profile, with nutritional deficiencies, pro-inflammatory status, and cardiometabolic risk in both study groups (Table 2).

### 2.4. Correlation Between Nutrient Intake, Clinical Variables, and Global DNA Methylation Percentage in Both Study Groups

**Figure 2:**
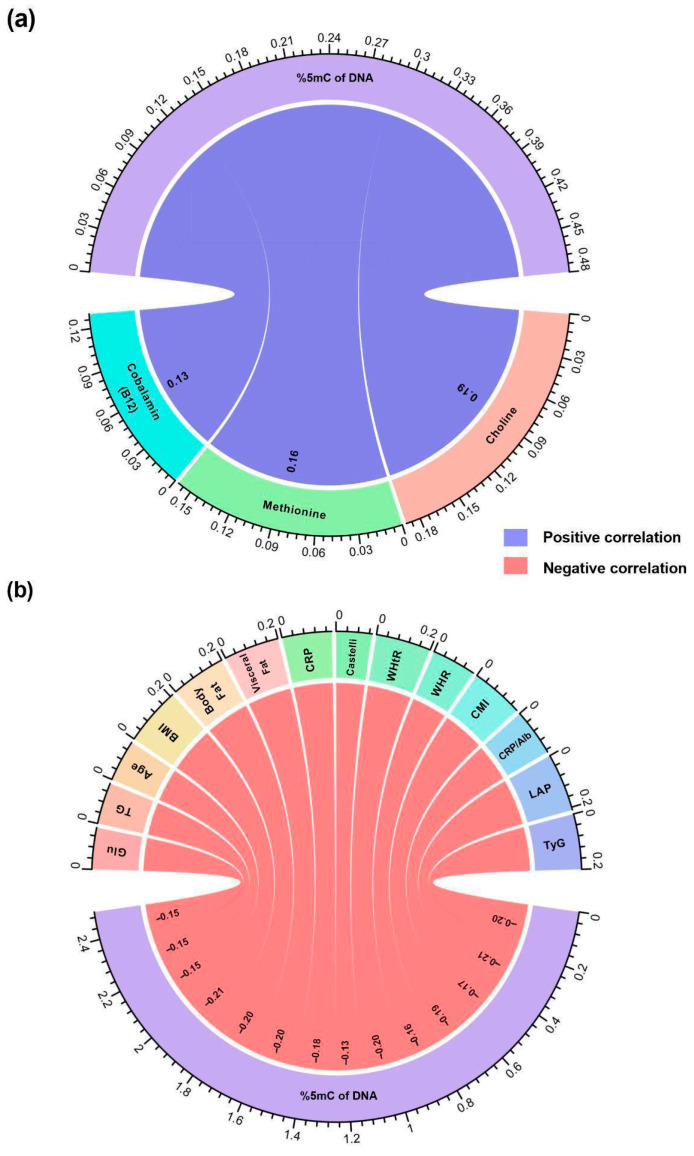
Correlations between methyl-donor nutrient intake, global DNA methylation percentage, and cardiometabolic variables in both study groups. (**a**) Correlogram of nutrient intake with global DNA methylation percentage in both study groups. Spearman correlation coefficients are represented as lines connecting global methylation percentage (purple) and nutrient intake (rainbow). Red line = negative correlation and blue line = positive correlation. (**b**) Correlogram of global DNA methylation percentage with cardiometabolic variables in both study groups. Spearman correlation coefficients are represented as lines connecting the cardiometabolic variables (rainbow) and the global methylation percentage (purple). Only correlations with a *p*-value < 0.05 are shown. Red line = negative correlation and blue line = positive correlation. Correlation coefficient obtained by the Spearman test. Glu: glucose; TG: triglycerides; BMI: body mass index; CRP: C-reactive protein; WHR: waist-to-hip ratio; WHtR: Waist-to-Height Ratio; CMI: cardiometabolic index (triglycerides/HDL-C); CRP/Alb: C-reactive protein/albumin ratio; LAP: lipid accumulation product; TyG: triglyceride-glucose index.

Regarding correlation analyses between nutrient intake and global DNA methylation percentage, a significant positive correlation was observed in the main methyl group donor nutrients. This suggests that a higher intake of choline (r = 0.19; *p* = 0.003), methionine (r = 0.16; *p* = 0.009), and cobalamin (B12) (r = 0.13; *p* = 0.04) is related to higher percentages of global leukocyte DNA methylation (Figure 2a).

Across clinical and cardiometabolic variables, a negative correlation was observed with the global methylation percentage. This relationship showed that as DNA methylation decreases, higher values are presented in metabolic parameters and age, specifically glucose (r = −0.15; *p* = 0.01), triglycerides (r = −0.15; *p* = 0.01), and age (r = −0.15; *p* = 0.01), as well as in anthropometric indicators such as BMI (r = −0.21; *p* < 0.001), body fat (r = −0.20; *p* = 0.001), and visceral fat (r = −0.20; *p* = 0.002). Similarly, significant correlations were observed with inflammatory markers and indexes, including CRP (r = −0.18; *p* = 0.004), the Castelli index (r = −0.13; *p* = 0.03), WHtR (r = −0.20; *p* = 0.001), and WHR (r = −0.16; *p* = 0.01), followed by CVD risk scores such as the CMI score (r = −0.19; *p* = 0.002), the CRP/albumin ratio (r = −0.17; *p* = 0.007), the LAP score (r = −0.21; *p* < 0.001), and the TyG index score (r = −0.20; *p* = 0.001) (Figure 2b).

### 2.5. Exploratory Association of Global DNA Hypomethylation with Disease Status, Nutritional Intake, and Cardiometabolic Risk Factors

Subsequently, we evaluated the potential association between nutrient intake deficiency, cardiometabolic risk factors, and the presence of DNA hypomethylation (<4.87%) in both study groups. These associations were analyzed using multivariate logistic regression models, adjusted for age, BMI, and the interaction between these two variables.

Initially, the association between the presence of RA and the risk of presenting global DNA hypomethylation was evaluated. It was observed that RA was significantly associated with hypomethylation in the unadjusted model (OR = 2.91; 95% CI: 1.7–5.1; *p* < 0.001) and remained significant in the model adjusted for BMI (OR = 2.1; 95% CI: 1.2–3.8; *p* = 0.01); however, this association lost statistical significance in the model adjusted for age (OR = 1.7; 95% CI: 0.9–3.2; *p* = 0.1) and in the combined model adjusted for age and BMI (OR = 1.5; 95% CI: 0.8–3; *p* = 0.19); suggesting that the relationship between the disease hypomethylation could be influenced by age and body composition (Table 3).

Regarding nutritional variables, it was observed that methionine intake showed a significant association with global DNA hypomethylation status in the unadjusted model (OR = 1.82; 95% CI: 1.05–3.15; *p* = 0.03) and remained significant when adjusting for BMI (OR = 1.9; 95% CI: 1.06–3.3; *p* = 0.03) (Table 3).

According to cardiometabolic risk indexes, it was observed that global DNA hypomethylation status was associated with high hs-CRP risk within the unadjusted model (OR = 2.9; 95% CI: 1.63–4.93; *p* < 0.001) and maintained its significance in the model adjusted for BMI (OR = 1.9; 95% CI: 1–3.6; *p* = 0.03) (Table 3). Similarly, global DNA hypomethylation status was associated with elevated waist circumference only in the unadjusted model (OR = 2.87; 95% CI: 1.65–5; *p* < 0.001), while excess weight evaluated by BMI was significantly associated in the unadjusted model (OR = 3.7; 95% CI: 2.12–6.51; *p* < 0.001) and in the one adjusted for age (OR = 2.3; 95% CI: 1.2–4.47; *p* = 0.01). Similarly, this pattern was observed to have a risk body fat percentage only in the unadjusted model (OR = 3; 95% CI: 1.67–5.3; *p* < 0.001) (Table 3).

Continuing with the cardiometabolic indexes, the global DNA hypomethylation status was associated with CMI risk score in all evaluated models: unadjusted model (OR = 4.5; 95% CI: 2.6–7.9; *p* < 0.001), model adjusted for age (OR = 3.14; 95% CI: 1.7–5.9; *p* < 0.001), model adjusted for BMI (OR = 3.5; 95% CI: 1.8–6.8; *p* < 0.001), and in the model adjusted for age and BMI (OR = 2.9; 95% CI: 1.5–5.9; *p* = 0.002). Similarly, the low DNA methylation status was associated with risk score by LAP index in the unadjusted model (OR = 3.6; 95% CI: 2–6.2; *p* < 0.001), in the one adjusted for age (OR = 2.1; 95% CI: 1.1–4.2; *p* = 0.03), and in the model adjusted for BMI (OR = 2.6; 95% CI: 1.2–5.6; *p* = 0.01). Additionally, a similar pattern was observed with the WHtR index risk score in the unadjusted model (OR = 3.16; 95% CI: 1.8–5.52; *p* < 0.001), the CRP/Albumin ratio risk score (OR = 2.46; 95% CI: 1.43–4.22; *p* = 0.001), for the TyG index risk score in the unadjusted model (OR = 4.1; 95% CI: 2.4–7.2; *p* < 0.001), adjusted for age (OR = 2.6; 95% CI: 1.4–5; *p* = 0.004), adjusted for BMI (OR = 3.2; 95% CI: 1.72–5.9; *p* < 0.001), and in the model adjusted for age and BMI (OR = 2.5; 95% CI: 1.2–4.8; *p* = 0.008). Finally, the low DNA methylation status was associated with the VAI index risk score in the unadjusted model (OR = 3.3; 95% CI: 1.9–5.8; *p* < 0.001), adjusted for age (OR = 2.2; 95% CI: 1.12–4.1; *p* = 0.01), adjusted for BMI (OR = 2.4; 95% CI: 1.3–4.4; *p* = 0.005), and in the model adjusted for age and BMI (OR = 2; 95% CI: 1.1–3.8; *p* = 0.03) (Table 3).

### 2.6. Associations Between Disease Status, Nutritional Intake, Cardiometabolic Risk Factors, and Low Global DNA Methylation

Linear regression analyses were performed to determine the effects of clinical, nutritional, cardiometabolic variables, and global leukocyte DNA methylation percentage, analyzed as a continuous variable in both study groups.

Initially, it was observed that the presence of RA was negatively associated with global DNA methylation levels in the unadjusted model (β = −5.7; 95% CI: −8.6 to −2.81; *p* < 0.001), maintaining its significance in the model adjusted for age (β = −3.8; 95% CI: −7.12 to −0.47; *p* = 0.02), adjusted for BMI (β = −4.75; 95% CI: −7.9 to −1.6; *p* = 0.003), and in the model adjusted for age and BMI (β = −3.6; 95% CI: −7 to −0.2; *p* = 0.04) (Table 4).

According to nutritional variables, it was identified that methionine intake showed a significant negative association with continuous global DNA methylation levels in all evaluated models: unadjusted model (β = −4.09; 95% CI: −7.3 to −0.88; *p* = 0.01), adjusted for age (β = −3.4; 95% CI: −6.5 to −0.22; *p* = 0.04), adjusted for BMI (β = −4.14; 95% CI: −7.31 to −0.97; *p* = 0.01), and in the model adjusted for age and BMI (β = −3.6; 95% CI: −6.77 to −0.41; *p* = 0.03). Similarly, pantothenic acid (B5) was negatively associated in the unadjusted model (β = −5.38; 95% CI: −9 to 1.78; *p* = 0.004), in those adjusted for age (*p* = 0.01) and for BMI (*p* = 0.01), but not in the model adjusted for age and BMI (β = −4.47; 95% CI: −8.08 to −0.85; *p* = 0.1) (Table 4).

Regarding cardiometabolic risk variables, it was observed that body fat was significantly associated with lower continuous global DNA methylation in the unadjusted model (β = 5.57; 95% CI: 3.26 to 7.87; *p* < 0.001), adjusted for age (β = 2.6; 95% CI: 0.5 to 4.69; *p* = 0.01), and adjusted for BMI (β = 1.57; 95% CI: 0.32 to 2.82; *p* = 0.01). Continuing with the indexes, the Castelli index showed association only in the unadjusted model (β = 0.34; 95% CI: 0.05 to 0.63; *p* = 0.02), a similar pattern to that observed in the triglycerides/HDL-C ratio (β = 0.37; 95% CI: 0.05 to 0.69; *p* = 0.02) and in the CMI score (β = 0.28; 95% CI: 0.08 to 0.48; *p* = 0.006). Likewise, the LAP score was significantly associated in the unadjusted model (β = 11.32; 95% CI: 4.14 to 18.5; *p* = 0.002). The WHR showed association in the unadjusted model (β = 0.03; 95% CI: 0.003 to 0.05; *p* = 0.03), as the WHtR (β = 0.05; 95% CI: 0.02 to 0.08; *p* = 0.002). Subsequently, it was identified that the TyG index was associated in the unadjusted model (β = 0.32; 95% CI: 0.18 to 0.47; *p* < 0.001), adjusted for age (β = 0.15; 95% CI: 0.01 to 0.28; *p* = 0.03), and adjusted for BMI (β = 0.19; 95% CI: 0.06 to 0.32; *p* = 0.005). CRP showed association only in the unadjusted model (β = 2.64; 95% CI: 0.22 to 5.06; *p* = 0.03). Finally, the VAI score presented a significant association in all models: unadjusted (β = 0.3; 95% CI: 0.15 to 0.40; *p* < 0.001), adjusted for age (β = 0.17; 95% CI: 0.05 to 0.3; *p* = 0.006), adjusted for BMI (β = 0.19; 95% CI: 0.07 to 0.31; *p* = 0.002), and in the model adjusted for age and BMI (β = 0.15; 95% CI: 0.3 to 0.27; *p* = 0.02) (Table 4).

## 3. Discussion

Altered DNA methylation patterns have been consistently identified in RA patients. RA synovial fibroblasts exhibit global DNA hypomethylation, a feature that has been linked to aberrant gene expression and aggressive cellular phenotypes [29]. These findings support the relevance of epigenetic dysregulation in RA pathophysiology; however, the factors influencing global DNA hypomethylation and its clinical implications remain incompletely understood. In this context, the present study aimed to evaluate the associations between dietary methyl-donor intake, global leukocyte DNA methylation levels, and cardiometabolic risk in RA patients and CS.

Our results indicate that RA status is associated with lower levels of global leukocyte DNA methylation compared with CS. Notably, when DNA methylation was analyzed as a continuous variable, RA status remained independently associated with lower methylation levels after adjustment for age and BMI, suggesting that RA-related epigenetic alterations persist beyond the influence of major demographic and anthropometric factors. In contrast, when DNA methylation was examined as a categorical variable, the association between RA and hypomethylation was attenuated in adjusted models by age and BMI, highlighting the greater sensitivity of continuous-variable analyses for detecting subtle yet biologically relevant epigenetic differences. It should be noted that the categorical classification was applied as an exploratory data-driven approach in the absence of established population-based reference values for global leukocyte DNA methylation.

RA pathogenesis arises from the interaction between genetic susceptibility, epigenetic regulation, and environmental exposures [30]. Within this framework, we explored the relationship between global leukocyte DNA methylation levels and dietary methyl-donor intake. We observed positive correlations between DNA methylation levels and the intake of choline, methionine, and cobalamin, while methionine intake remained independently associated with global DNA methylation in adjusted linear models. These findings are biologically plausible, as DNA methylation depends on one-carbon metabolism and the availability of S-adenosyl-methionine, which in turn relies on adequate intake of methyl-donor nutrients such as methionine, choline, and B vitamins [31]. Although the association between methionine intake and categorical hypomethylation did not reach statistical significance in fully adjusted logistic models, the consistency observed in continuous analyses supports a modest but independent relationship between dietary methyl-donor intake and global epigenetic status.

In contrast, several cardiometabolic variables, including glucose, triglycerides, body mass index, CRP, and composite cardiometabolic indexes, were inversely correlated with global DNA methylation levels. Notably, age was also negatively correlated with DNA methylation, consistent with previous evidence demonstrating age-related epigenomic remodeling across genomic compartments [32]. For this reason, age-adjusted models were prioritized in multivariable analyses. Collectively, these findings support the concept that global leukocyte DNA methylation levels reflect cumulative metabolic and inflammatory burden rather than isolated disease-specific effects.

The observed correlations between lower DNA methylation levels and unfavorable body composition measures such as increased BMI, waist circumference, and body fat percentage support the link between epigenetic regulation and cardiometabolic health. Lower DNA methylation levels were consistently associated with higher cardiometabolic risk scores and elevated inflammatory markers across unadjusted and adjusted models, particularly in continuous and association analyses, the VAI score was associated, which has recently proven to be an indicator of adipose distribution and function that indirectly expresses cardiometabolic risk [33]. Chronic low-grade inflammation is recognized as a central mechanism linking obesity to cardiometabolic disorders, and inflammatory mediators have been shown to induce epigenetic alterations, including changes in DNA methylation [34]. Additionally, DNA methylation plays a functional role in monocyte-to-macrophage differentiation and macrophage polarization, processes that may influence the proinflammatory status and cardiometabolic risk [35]. Previous studies have also reported hypomethylation of genes such as *LEP* and repetitive elements like LINE-1 in association with obesity, dyslipidemia, and adverse lipid profiles [36]. Although gene-specific methylation was not assessed in the present study, these findings provide a biological context supporting the observed associations at the global methylation level.

Although multiple cardiometabolic indexes were evaluated, these indexes capture closely related dimensions of adiposity, dyslipidemia, inflammation, and insulin resistance rather than independent outcomes. Importantly, the associations between global leukocyte DNA methylation levels and cardiometabolic risk, such as the VAI score, were consistent in direction and magnitude across conceptually related indexes, particularly in continuous multivariable models. This internal consistency supports the presence of a shared cardiometabolic phenotype associated with lower DNA methylation levels, rather than isolated false-positive findings due to multiple testing. Nevertheless, these results should be interpreted as complementary rather than independent signals and warrant confirmation in longitudinal studies.

Therefore, according to our findings, the strengths of this study should be highlighted. First, the assessment of global leukocyte DNA methylation levels as a continuous variable allowed us to capture subtle but biologically meaningful epigenetic variation, providing greater sensitivity than categorical approaches. Second, the integrative evaluation of dietary methyl-donor intake, epigenetic status, and multiple cardiometabolic indices offers a comprehensive perspective on the interplay between nutrition, inflammation, and metabolic health in RA and CS. Third, the inclusion of both RA patients and CS enabled the identification of shared and disease-related epigenetic patterns, supporting the interpretation of global DNA methylation as a biomarker of systemic inflammatory and metabolic burden rather than an isolated disease-specific alteration. Finally, the multivariable models adjusted for key confounders such as age and BMI strengthen the robustness and interpretability of the observed associations.

Despite this, several limitations should be acknowledged. First, the cross-sectional design precludes causal inference, and reverse causation between inflammation, cardiometabolic alterations, and DNA methylation cannot be excluded. Second, the lack of detailed adjustment for pharmacological treatment, particularly methotrexate, represents a potential source of residual confounding. Regarding pharmacological treatment, it represents an additional factor that may influence DNA methylation patterns in RA. Previous evidence indicates that methotrexate therapy can partially reverse global DNA hypomethylation in RA patients, potentially contributing to its anti-inflammatory effects [17]. Given that most patients in our study were receiving methotrexate, its influence cannot be excluded and may have attenuated disease-related differences in DNA methylation. Methodological limitations that we could mention are that the global leukocyte DNA methylation ELISA kit captures an average methylation signal across multiple genomic regions and is influenced by cumulative inflammatory, metabolic, and environmental exposures. Furthermore, global DNA methylation levels were measured in total peripheral blood leukocytes rather than in specific immune cell subsets. Given that leukocyte populations differ in their methylation profiles and that RA and cardiometabolic alterations may influence leukocyte composition, part of the observed variability could reflect changes in cell-type distribution. Thus, our results should be interpreted as integrated systemic epigenetic signatures rather than cell-specific effects. Nevertheless, the persistence of significant associations in continuous models suggests that global leukocyte DNA methylation levels reflect a complex integration of disease-related, metabolic, and environmental influences rather than treatment effects alone.

Even considering these limitations, this study provides evidence supporting an association between dietary methyl-donor intake, global leukocyte DNA methylation levels, and cardiometabolic risk in both RA patients and CS. From an integrative perspective, global DNA methylation levels may represent a useful epigenetic biomarker reflecting the interplay between nutrition, inflammation, and cardiometabolic health in chronic inflammatory diseases. Nonetheless, future studies should incorporate longitudinal designs, pharmacotherapy stratification, and gene or immune cell subpopulations’ specific epigenetic analyses, as well as additional epigenetic mechanisms such as noncoding RNAs and histone modifications, to further elucidate the complex role of epigenetic regulation in RA and its cardiometabolic comorbidities.

## 4. Materials and Methods

### 4.1. Participants

A cross-sectional study was conducted with 253 females divided into groups: 123 RA patients and 130 CS. RA patients were classified according to the ACR/EULAR criteria [37,38], and the clinical disease activity was assessed by the 28-joint Disease Activity Score using C-reactive protein [DAS-28 (CRP)] criteria [39]. RA patients were recruited from 2023 to 2025 in the Rheumatology Department of the Hospital Civil Fray Antonio Alcalde in Guadalajara, Jalisco, Mexico. RA patients were included regardless of disease activity status (remission, low, moderate, or high activity) and ongoing pharmacological treatment at the time of recruitment, provided that no overlapping autoimmune diseases were present. CS were recruited from the Guadalajara Metropolitan Area and were women of similar age who reported no personal history of autoimmune or chronic allergic diseases, no current or previous use of anti-inflammatory, hormonal, or hormonal contraceptive treatments, and no family history of autoimmune diseases among parents, siblings, or grandparents. For both study groups, inclusion criteria were age ≥ 18 years, female sex, and Mexican mestizo ancestry, defined as residence in the Western region of Mexico for at least three generations. All participants provided written informed consent before inclusion in the study. Exclusion criteria for both groups included pregnancy, current or previous diagnosis of malignant disease, diagnosis of human immunodeficiency virus or viral hepatitis, and the presence of acute or recent infection at the time of recruitment.

### 4.2. Ethical Considerations

This cross-sectional study was approved by the Research Ethics Committee of the Hospital Civil Fray Antonio Alcalde (Approval Code: CEI. 135/23) on 4 May 2023, in accordance with international research ethics guidelines. All participants signed an informed consent.

### 4.3. Biochemical Evaluation

For biochemical evaluation, a blood sample was obtained from each participant from antecubital venipuncture collected in the morning between 7:30 and 10:00 a.m. after an overnight fast (12 h) and then centrifuged for 10 min to obtain serum. Serum metabolites like glucose (Cat. 11504, BioSystems^®^ kits, Barcelona, Spain) was assessed by the glucose oxidase/peroxidase colorimetric method, triglycerides (Cat. 12528, BioSystems^®^ kits, Barcelona, Spain) by the glycerol phosphate oxidase/peroxidase colorimetric method, total cholesterol (Cat. 11805, BioSystems^®^ kits, Barcelona, Spain), by the cholesterol oxidase/peroxidase colorimetric method, high-density lipoprotein-cholesterol (HDL-C) (Cat. 11557, BioSystems^®^ kits, Barcelona, Spain) by the detergent colorimetric method, low-density lipoprotein-cholesterol (LDL-C) (Cat. 11585, BioSystems^®^ kits, Barcelona, Spain) by the detergent colorimetric method, C-reactive protein (CRP) (Cat. 31927, BioSystems^®^ kits, Barcelona, Spain) by the latex-high sensitivity turbidimetric method, uric acid (Cat. 11821, BioSystems^®^ kits, Barcelona, Spain) by the uricase/peroxidase colorimetric method, and albumin (Cat. 21547, BioSystems^®^ kits, Barcelona, Spain) by the bromocresol green colorimetric method. All analytes were assessed by a Clinical Chemistry Analyzer (Mindray-BS-240, Shenzhen, China). For all determinations, the biochemical control serum levels I (cat. 18005) and II (cat. 18007) were used to verify the accuracy of the measurement procedure. (BioSystems^®^ kits, Barcelona, Spain).

### 4.4. Nutritional Status and Dietary Intake

The analysis of nutrient intake was performed with the Nutritionist Pro Diet software v7.5 (Axxya Systems, Washington, DC, USA) based on three 24 h food records (two weekdays and one weekend day) performed in a personal interview carried out by a trained nutritionist and for a more accurate quantitative estimation of the food that participants ingested in each of their mealtimes, they were asked for quantity, type or variety and the additives used in the meals preparation, with the support of the “Mexican food photograph album” validated for the visual estimation of food in the Mexican population according to our standardized protocol reported in our previous study by Meza-Meza et al. [40]. Adequate consumption of macronutrients was considered according to the recommended distribution range for the Mexican population [41]: carbohydrates (55–63%), proteins (15–18%), lipids (25–30%), and saturated fat (<7%) adjusted by the non-resting total expenditure of each participant. The adequacy achievement of each micronutrient was obtained and classified according to the dietary reference intakes (DRI) described in the NOM-051-SCFI/SSA1-2010-MEX as follows: average nutrient consumption × 100/DRI average nutrient intake, concerning the recommendation of nutrient intake for the Mexican population [42]. For nutrients lacking officially established cut-off values in the Mexican population (e.g., choline and betaine), population-specific tertiles were constructed and used as exploratory thresholds to explore potential associations with global DNA methylation and cardiometabolic risk markers.

### 4.5. Body Composition and Anthropometric Evaluation

Body composition evaluation: weight (kg), muscle mass (kg), and fat mass (%) were performed through bioimpedance analysis prediction method (TANITA^®^ Ironman™ body composition Monitor BC-549, Arlington Heights, IL, USA), and height was measured to the nearest 0.1 cm using a stadiometer (Seca, Hamburg, Germany). Waist and hip circumferences were measured twice using a flexible metal tape with an accuracy of ±0.1 cm (Lufkin^®^ executive thinline W606, ME, USA), with the subject standing with feet together and arms crossed. Waist circumference (WC) was measured at the midpoint between the costal margin and iliac crest in the mid-axillary line in a standing position at the end of a gentle expiration, and hip circumference measurement was taken around the widest portion of the buttocks [43]. Body mass index (BMI) was calculated [BMI = (weight, kg)/(height, m^2^)] according to the NOM-043-SSA2-2012-MEX based on World Health Organization (WHO) criteria [32]. The waist-to-hip ratio (WHR) was calculated; the waist-to-height ratio (WHtR) was calculated [WHtR = (waist, cm)/(height, cm)] according to our previous study by Meza-Meza et al. [40].

### 4.6. Cardiometabolic Risk Assessment

Cardiometabolic risk was assessed using a panel of validated anthropometric, biochemical, and composite cardiometabolic indexes that reflect adiposity, dyslipidemia, insulin resistance, and systemic inflammation. These included the Castelli index, Kannel index, triglyceride/high-density lipoprotein cholesterol ratio (TG/HDL-C), cardiometabolic index (CMI), lipid accumulation product (LAP), waist-to-height ratio (WHtR), triglyceride-glucose index (TyG), visceral adiposity index (VAI), CRP/albumin ratio, and the Framingham risk score.

Cardiometabolic indexes were calculated and interpreted according to our previous study by Campos-López et al., and Pesqueda-Cendejas et al. [44,45]. For this study we applied the following cardiometabolic indexes: (a) the Castelli index classified as CVD risk an score of ≥4.5; (b) the Kannel index was classified as high CVD risk an score of ≥3; (c) the TG/HDL-C ratio was classified as high CVD risk an score ≥ 3, (d) the CMI x classified by tertiles, interpreted the highest T3rd as high CVD risk (≥0.61), (e) the LAP score x was also classified by tertiles and the T2nd (≥10.74) and interpreted as CVD risk, (f) CRP serum levels interpreted as CVD risk with a value ≥ 1 mg/dL of CRP based on the criteria of the centers for disease control and prevention and the American heart association, (g) The WHR classified as risk with a score ≥ 0.85); (h) the WHtR with a score ≥ 0.5 classified as a risk for metabolic abnormalities, according to the WHO cutoff values. [43]. (i) Framingham Index taken with a risk value ≥ 10% of probability of atherosclerotic cardiovascular disease [46], (j) CRP/Albumin ratio classified by tertiles, interpreted the highest T3rd as high CVD risk with a score > 0.320 [47], (k) TyG (ln[(fasting triglycerides (mg/dL) × fasting glucose (mg/dL))/2]) classified by tertiles [48], interpreted the highest T2nd as high CVD risk with a score ≥ 8.01, (l) VAI score, (Waist circumference (cm)/(36.58 + (1.89 × BMI))) × (Triglycerides (mmol/L)/0.81) × (1.52/HDL-C (mmol/L)) classified by tertiles, interpreted the highest T3rd as high CVD risk [33].

### 4.7. Global Leukocyte DNA Methylation Quantification and Hypomethylation Status Definition

Genomic DNA (gDNA) was extracted from peripheral blood leukocytes using the salting-out method [49]; Global DNA methylation was quantified using a commercially available 5-mC DNA ELISA kit (Cat. D5325, Zymo Research, CA, USA), following the manufacturer’s instructions. Briefly, 100 ng of purified gDNA per sample were denatured to single-stranded DNA, immobilized onto assay wells, and incubated with a monoclonal anti-5-methylcytosine antibody, followed by HRP-conjugated secondary antibody detection. Absorbance was measured at 405–450 nm using an ELISA plate reader. All samples and standards were analyzed in duplicate. Quantification of global DNA methylation was performed using a standard curve generated from mixtures of fully methylated and unmethylated control DNA provided by the manufacturer, allowing estimation of the relative percentage of 5-mC in each sample. According to kit specifications, the assay reliably detects ≥0.5% 5-mC per 100 ng of input DNA. This ELISA-based method provides an estimate of average global DNA methylation across the genome and has been shown to correlate with mass spectrometry–based measurements; however, it does not capture locus-specific methylation patterns or cell-type–specific heterogeneity within leukocyte populations. To define hypomethylation status, global leukocyte DNA methylation percentages from both study groups were stratified into tertiles. Then, the second tertile was used as the cut-off value, classifying hypomethylation as <4.87% and hypermethylation as ≥4.87% global leukocyte DNA methylation. This categorization was applied as an exploratory data-driven approach, given the absence of established human population-based reference values for global leukocyte DNA methylation levels.

### 4.8. Statistical Analysis

Statistical analyses were performed using STATA version 15 (StataCorp, College Station, TX, USA) and GraphPad Prism version 8.0 (GraphPad Software, San Diego, CA, USA). Sample size and statistical power were estimated using the Fleiss formula for case–control studies [27]. The distribution of continuous variables was assessed using the Shapiro–Wilk test. As most variables showed a non-normal distribution, continuous data are presented as medians with 5th–95th percentiles, while categorical variables are expressed as frequencies and percentages. Between-group comparisons of categorical variables were conducted using the chi-square test. Differences between two independent groups for continuous non-normally distributed variables were evaluated using the Mann–Whitney U test. To examine associations between dietary methyl-donor intake, global leukocyte DNA methylation, and clinical and cardiometabolic variables, both linear and logistic regression models were applied. All regression models were adjusted for age and/or BMI as relevant confounders. A *p*-value < 0.05 was considered statistically significant for all analyses.

## 5. Conclusions

RA patients exhibited lower global leukocyte DNA methylation levels compared with CS. In both study groups, lower DNA methylation levels were consistently associated with low methionine intake and an unfavorable cardiometabolic profile.

## Figures and Tables

**Table 1:** Clinical characteristics of patients with RA and CS.

Variable	RA n = 123	CS n = 130	*p* Value
*Disease Status*			
Disease duration (years) ^a^	6 (0.8–23)	-	
Early RA (<1 year) ^b^	13 (15/119)	-	
Established RA (≥1 year) ^b^	87 (104/119)	-	
*Clinical Assessment*			
ESR (mm/h) ^a^	34 (10–93)	-	
C-reactive protein (mg/L) ^a^	4.6 (0.60–33)	0.87 (0–10.27)	**<0.001**
Anti-CCPs (U/mL) ^a^	214.35 (1.25–1051.45)	-	
RF (IU/mL) ^a^	96 (8–500)	-	
Swollen joints ^a^	0 (0–9)	-	
Tender joints ^a^	2 (0–10)	-	
DAS28 CRP ^a^	2.97 (1.33–5.43)		
Remission (<2.6) ^b^	37 (44/120)	-	
Active Disease (≥2.6) ^b^	63 (76/120)	-	
*Hematological Data*			
Hemoglobin (g/dL) ^a^	13.24 (9.41–15.30)	-	
Hematocrit (%) ^a^	40.43 (28.67–45.70)	-	
Erythrocytes (1 × 10^6^/µL) ^a^	4.39 (3.80–5.22)	-	
Leukocytes (1 × 10^3^/µL) ^a^	7.15 (4.59–12)	-	
Platelets (1 × 10^3^/µL) ^a^	272.5 (177.9–403)	-	
Lymphocytes (1 × 10^3^/µL) ^a^	1.96 (0.92–3.39)	-	
*Treatment*			
NSAIDs ^b^	69 (83/120)	-	
Glucocorticoids ^b^	22 (26/120)	-	
Sulfasalazine ^b^	69 (83/120)	-	
Chloroquine ^b^	36 (44/121)	-	
Methotrexate ^b^	84 (102/121)	-	

^a^ Values given as median (p5–p95); ^b^ Data presented as percentages (n/total evaluated patients). *p*-values highlighted in bold indicate statistical significance (*p* < 0.05). (-) Not determined for the CS group. RF: Rheumatoid factor; ESR: Erythrocyte Sedimentation Rate; CRP: C-Reactive Protein; Anti-CCP: Anti-cyclic citrullinated peptide antibodies; NSAIDs: Non-steroidal anti-inflammatory drugs.

**Table 2:** Comparison of the frequency of nutritional deficiencies, anthropometric alterations, and cardiometabolic risk factors according to DNA methylation status in both study groups.

Variables	Hypomethylation (<4.87%) n = 170	Hypermethylation (≥4.87%) n = 83	*p* Value
*Intake of methyl group donor nutrients*			
Pantothenic Acid (B5) deficient (<4 mg/day) ^a^	80 (125/157)	68 (55/81)	0.05
Cobalamin (B12) deficient (<2.4 µg/day) ^a^	24 (38/156)	21 (17/81)	0.50
Calcium deficient (<1000 mg/day) ^a^	63 (98/156)	56 (45/81)	0.28
Choline-deficient (<235.6 mg/day) ^a^	68 (107/158)	57 (47/83)	0.08
Methionine-deficient (<978.3 mg/day) ^a^	68 (108/158)	54 (45/83)	**0.03**
*Body composition*			
Waist Circumference (≥80 cm) ^a^	58 (97/167)	33 (27/83)	**<0.001**
Excess BMI (≥25 kg/m^2^) ^a^	62 (104/169)	30 (25/83)	**<0.001**
Excess Body Fat (≥31%) ^a^	50 (85/169)	25 (21/83)	**<0.001**
Excessive Visceral Fat (≥13%) ^a^	6 (10/170)	4 (3/82)	0.5
*Biochemical data*			
Altered Glucose (≥100 mg/dL) ^a^	24 (40/170)	8 (7/83)	**0.01**
High Cholesterol (≥200 mg/dL) ^a^	22 (37/169)	13 (11/83)	0.07
High HDL-C (>60 mg/dL) ^a^	20 (34/170)	33 (27/83)	0.08
Altered LDL-C (≥100 mg/dL) ^a^	46 (78/170)	31 (26/83)	**0.01**
High Triglycerides (≥150 mg/dL) ^a^	12 (19/170)	9 (8/83)	0.6
Hypoalbuminemia (<3.4 g/dL) ^a^	6 (10/170)	7 (6/83)	0.7
*Cardiometabolic indexes*			
Hs-CRP CVD Risk (≥1 mg/L) ^a^	75 (128/170)	52 (43/83)	**<0.001**
Castelli risk score (≥4.5) ^a^	21 (35/170)	12 (10/83)	0.06
Kannel risk score (≥3) ^a^	16 (27/170)	12 (10/83)	0.4
TG/HDL-C High risk score (≥3) ^a^	19 (33/170)	14 (12/83)	0.3
CMI Risk score (≥0.61) ^a^	74 (121/163)	39 (32/83)	**<0.001**
LAP High risk score (≥10.74) ^a^	30 (108/165)	36 (30/83)	**<0.001**
WHR Android risk (≥0.85) ^a^	39 (65/168)	24 (20/82)	**0.02**
WHtR High risk score (≥0.5) ^a^	59 (98/166)	31 (26/83)	**<0.001**
Framingham risk score (≥10%) ^a^	6 (5/87)	0 (0/17)	0.6
CRP/Albumin risk score (>0.320) ^a^	69 (118/170)	48 (40/83)	**0.004**
TyG risk score (≥8.01) ^a^	74 (126/170)	41 (34/83)	**<0.001**
VAI risk score (≥1.064) ^a^	72 (116/161)	44 (35/80)	**<0.001**

^a^ Data presented as percentages (n/total evaluated patients). *p*-values highlighted in bold indicate statistical significance (*p* < 0.05; BMI: Body Mass Index; HDL-C: High-Density Lipoprotein-cholesterol; LDL-C: Low-Density Lipoprotein-cholesterol; TG: Triglycerides; hs-CRP: high-sensitivity C-reactive protein; CMI: Cardiometabolic Index; LAP: Lipid Accumulation Product; WHR: Waist to Hip Ratio; WHtR: Waist to Height Ratio; TyG: Triglyceride Glucose Index; VAI: Visceral Adiposity Index.

**Table 3:** Exploratory associations between biochemical, body composition, cardiometabolic variables, nutrient intake, and low global leukocyte DNA methylation status in both study groups.

Variables	Stratification	Model 1 OR (CI 95%)	*p* Value	Model 2 OR (CI 95%)	*p* Value	Model 3 OR (CI 95%)	*p* Value	Model 4 OR (CI 95%)	*p* Value
*Associations between disease status and global leukocyte DNA hypomethylation*									
RA	Disease (Yes or no)	2.91 (1.7–5.1)	**<0.001**	1.7 (0.9–3.2)	0.1	2.1 (1.2–3.8)	**0.01**	1.5 (0.8–3)	0.2
*Associations between low intake of methyl group donor nutrients and global leukocyte DNA hypomethylation*									
Methionine	Low (<978.3 mg/day)	1.82 (1.05–3.15)	**0.03**	1.61 (0.9–2.85)	0.1	1.9 (1.06–3.3)	**0.03**	1.7 (0.94–3.03)	0.07
Pantothenic Acid (B5)	Deficient (<4 mg/day)	0.54 (0.3–1)	0.05	0.63 (0.33–1.19)	0.1	0.63 (0.33–1.18)	0.1	0.65 (0.34–1.3)	0.2
*Associations between global leukocyte DNA hypomethylation and cardiometabolic risk status*									
Hs-CRP	High risk (≥1 mg/L)	2.9 (1.63–4.93)	**<0.001**	1.75 (0.94–3.22)	0.08	1.9 (1–3.6)	**0.03**	1.54 (0.8–2.9)	0.2
Waist Circumference	Risk score (≥80 cm)	2.87 (1.65–5)	**<0.001**	1.47 (0.72–2.97)	0.3	1.58 (0.7–3.7)	0.29	0.93 (0.36–2.4)	0.8
BMI	Excess weight (≥25 kg/m^2^)	3.7 (2.12–6.51)	**<0.001**	2.3 (1.2–4.47)	**0.01**	-	-	-	-
Body Fat percentage	Excess weight (≥31%)	3 (1.67–5.3)	**<0.001**	1.75 (0.9–3.4)	0.1	1.64 (0.7–3.86)	0.2	1.3 (0.5–3.3)	0.5
CMI Score	High Risk (≥0.61)	4.5 (2.6–7.9)	**<0.001**	3.14 (1.7–5.9)	**<0.001**	3.5 (1.8–6.8)	**<0.001**	2.9 (1.5–5.9)	**0.002**
LAP Score	High Risk (≥10.74)	3.6 (2–6.2)	**<0.001**	2.1 (1.1–4.2)	**0.03**	2.6 (1.2–5.6)	**0.01**	1.8 (0.8–4.1)	0.16
WHtR Index	High Risk (≥0.5)	3.16 (1.8–5.52)	**<0.001**	1.7 (0.83–3.48)	0.1	2(0.86–4.54)	0.11	1.21 (0.48–3.06)	0.7
CRP/Albumin Ratio	High Risk (≥0.320)	2.46 (1.43–4.22)	**0.001**	1.55 (0.85–2.82)	0.1	1.6 (0.9–3)	0.1	1.3 (0.72–2.5)	0.4
Stratified TyG Index	High Risk (≥8.01)	4.1 (2.4–7.2)	**<0.001**	2.6 (1.4–5)	**0.004**	3.2 (1.72–5.9)	**<0.001**	2.5 (1.2–4.8)	**<0.01**
Stratified VAI Index	High Risk (≥1.065)	3.3 (1.9–5.8)	**<0.001**	2.2 (1.12–4.1)	**0.01**	2.4 (1.3–4.4)	**0.005**	2 (1.1–3.8)	**0.03**

Model 1: Unadjusted model; Model 2: Model adjusted for age; Model 3: Model adjusted for BMI; Model 4: Model adjusted for age and BMI. Data are presented as Odds Ratio (95% Confidence Interval). Logistic regression models were used to estimate the association with low global DNA methylation. *p*-values highlighted in bold indicate statistical significance (*p* < 0.05). RA: Rheumatoid Arthritis; BMI: Body Mass Index; hs-CRP: High Sensitivity C Reactive Protein; CMI: Cardiometabolic Index; LAP: Lipid Accumulation Product; WHtR: Waist to Height Ratio; TyG: Triglyceride Glucose Index; VAI: Visceral Adiposity Index; OR: Odds Ratio; CI: Confidence Interval.

**Table 4:** Associations of clinical, nutritional, and cardiometabolic variables on global leukocyte DNA methylation percentage.

Variables	Model 1	*p* Value	Model 2	*p* Value	Model 3	*p* Value	Model 4	*p* Value
*Association between disease status and global leukocyte DNA methylation levels*								
Global leukocyte DNA Methylation levels	−5.7 (−8.6 to −2.81)	**<0.001**	−3.8 (−7.12 to −0.47)	**0.02**	−4.75 (−7.9 to −1.6)	**0.003**	−3.6 (−7 to −0.2)	**0.04**
*Association between the low Intake of methionine (<978.3 mg/day) and global leukocyte DNA methylation levels*								
Global leukocyte DNA Methylation levels	−4.09 (−7.3 to −0.88)	**0.01**	−3.4 (−6.5 to −0.22)	**0.04**	−4.14 (−7.31 to −0.97)	**0.01**	−3.6 (−6.77 to −0.41)	**0.03**
*Association between the low Intake of pantothenic acid (<4 mg/day) and global leukocyte DNA methylation levels*								
Global leukocyte DNA Methylation levels	−5.38 (−9 to 1.78)	**0.004**	−4.63 (−8.18 to −1.08)	**0.01**	−4.72 (−8.36 to −1.07)	**0.01**	−4.47 (−8.08 to −0.85)	0.1
*Associations between low global leukocyte DNA methylation (<4.87%) and cardiometabolic risk scores*								
Body fat (kg)	5.57 (3.26 to 7.87)	**<0.001**	2.6 (0.5 to 4.69)	**0.01**	1.57 (0.32 to 2.82)	**0.01**	1.06 (−0.74 to 2.31)	0.09
Castelli Index (Chol/HDL-C)	0.34 (0.05 to 0.63)	**0.02**	0.15 (−0.14 to 0.44)	0.3	0.23 (−0.6 to 0.52)	0.1	0.12 (−0.17 to 0.43)	0.4
TG/HDL-C ratio	0.37 (0.05 to 0.69)	**0.02**	0.09 (−0.22 to 0.4)	0.5	0.16 (−0.15 to 0.47)	0.3	0.03 (−0.27 to 0.34)	0.8
CMI Index (TG/HDL-C)	0.28 (0.08 to 0.48)	**0.006**	0.07 (−0.11 to 0.27)	0.4	0.07 (−0.1 to 0.25)	0.4	0.006 (−0.17 to 0.18)	0.9
LAP score	11.32 (4.14 to 18.5)	**0.002**	2.73 (−3.86 to 9.34)	0.4	−1.15 (−6.1 to 3.8)	0.7	−1.15 (−6.1 to 3.8)	0.6
WHR Index score	0.03 (0.003 to 0.05)	**0.03**	−0.006 (−0.03 to 0.017)	0.6	0.005 (−0.02 to 0.03)	0.6	−0.01 (−0.03 to 0.01)	0.3
WHtR Index score	0.05 (0.02 to 0.08)	**0.002**	0.01 (−0.01 to 0.04)	0.3	0.001 (−0.02 to 0.02)	0.9	−0.004 (−0.02 to 0.01)	0.7
TyG Index score	0.32 (0.18 to 0.47)	**<0.001**	0.15 (0.01 to 0.28)	**0.03**	0.19 (0.06 to 0.32)	**<0.01**	0.11 (−0.01 to 0.24)	0.08
CRP (mg/dL)	2.64 (0.22 to 5.06)	**0.03**	1.7 (−0.8 to 4.22)	0.2	1.57 (−0.87 to 4.01)	0.2	1.32 (−1.17 to 3.83)	0.3
VAI Index score	0.3 (0.15 to 0.40)	**<0.001**	0.17 (0.05 to 0.3)	**<0.01**	0.19 (0.07 to 0.31)	**0.002**	0.15 (0.3 to 0.27)	**0.02**

Model 1: Unadjusted model; Model 2: Model adjusted for age; Model 3: Model adjusted for BMI; Model 4: Model adjusted for age and BMI. Data are presented as Beta coefficient (β; 95% Confidence Interval). Linear regression models were used to assess the effect of variables on global DNA methylation percentage. *p* values highlighted in bold indicate statistical significance (*p* < 0.05). BMI: Body Mass Index; TG: Triglycerides; Chol: Cholesterol; HDL: High-Density Lipoprotein; CMI: Cardiometabolic Index; LAP: Lipid Accumulation Product; WHtR: Waist-to-Height Ratio; TyG: Triglyceride-Glucose Index; VAI: Visceral Adiposity Index.

## Data Availability

The original contributions presented in this study are included in the article. Further inquiries can be directed to the corresponding authors.

## Abbreviations

ACPA	Anti-citrullinated peptide antibodies
BMI	Body mass index
CMI	Cardiometabolic index
CRP	C-reactive protein
CS	Control subjects
CVD	Cardiovascular disease
DAS28	Disease activity score in 28 joints
ESR	Erythrocyte sedimentation rate
HDL-C	High-density lipoprotein-cholesterol
hs-CRP	High-sensitivity C-reactive protein
IL-6	Interleukin-6
LAP	Lipid accumulation product
LDL-C	Low-density lipoprotein-cholesterol
MMP-1	Matrix metalloproteinase-1
NSAIDs	Non-steroidal anti-inflammatory drugs
RA	Rheumatoid arthritis
RF	Rheumatoid factor
SAM	S-adenosylmethionine
STAT3	Signal transducer and activator of transcription 3
TBX5	T-box transcription factor 5
TyG	Triglyceride-glucose index
VAI	Visceral adiposity index
WC	Waist circumference
WHR	Waist-to-hip ratio
WHtR	Waist-to-height ratio
5-mC	5-methylcytosine
